# O.5.3-4 Key components in the implementation of a co-produced framework for community-based physical activity promotion: a comparative case study of six diverse pilot communities

**DOI:** 10.1093/eurpub/ckad133.255

**Published:** 2023-09-11

**Authors:** Lea Dippon, Natalie Helsper, Simone Kohler, Philipp Weber, Leonie Birkholz, Alfred Rütten, Klaus Pfeifer, Jana Semrau

**Affiliations:** Friedrich-Alexander-Universität Erlangen-Nürnberg; lea.j.dippon@fau.de; Friedrich-Alexander-Universität Erlangen-Nürnberg; lea.j.dippon@fau.de; Friedrich-Alexander-Universität Erlangen-Nürnberg; lea.j.dippon@fau.de; Friedrich-Alexander-Universität Erlangen-Nürnberg; lea.j.dippon@fau.de; Friedrich-Alexander-Universität Erlangen-Nürnberg; lea.j.dippon@fau.de; Friedrich-Alexander-Universität Erlangen-Nürnberg; lea.j.dippon@fau.de; Friedrich-Alexander-Universität Erlangen-Nürnberg; lea.j.dippon@fau.de; Friedrich-Alexander-Universität Erlangen-Nürnberg; lea.j.dippon@fau.de

## Abstract

**Purpose:**

As part of the project KOMBINE, we co-produced a framework with nine key components for community-based physical activity promotion (c-PAP) focusing on health equity together with stakeholders from policy, practice and research. We then implemented the framework in six diverse pilot communities (PC). The aim of this study is to analyse process and output of the participatory implementation.

**Methods:**

The framework includes six phases: (1) preparation, (2) assessment, (3) setting up cooperative planning groups including steering committees (4) organization of the planning process, (5) development and (6) implementation of measures. We used an online questionnaire and meeting minutes to evaluate the process by means of qualitative content analysis. We then compared the PC regarding the four key components *intersectoral collaboration, participation, political support and resources*. For the output evaluation, we conducted a document analysis of the action plans from phase 5.

**Results:**

Each PC established a cooperative planning group with *intersectoral collaboration*. The *participation* of people with social disadvantages proved to be challenging, but was more successful in rural compared to urban PC. Further, some PC had to invest many resources to ensure *political support* and had difficulties involving high-ranking political stakeholders, while in others *political support* was a given throughout the entire process. Personnel *resources* were a highly relevant topic in all PC, while financial *resources* played a bigger role in rural and socioeconomically deprived PC. The output was a broad range of measures (personal skills (n = 28), infrastructure (n = 70), policy action (n = 11), and community action (n = 3)) relevant to the specific needs of each PC.

**Conclusions:**

Through the analysis, we shed light on the interplay between context, process and output and the role of key components for the successful implementation of sustainable and equitable c-PAP in different contexts. Next steps are the evaluation of outcome and impact to inform the effective scale up of this approach.

**Funding Source:**

This work was supported by the Federal Centre of Health Education (BZgA) on behalf of and with funds from the statutory health insurances according to § 20a SGB V in the context of the GKV Alliance for Health (https://www.gkv-buendnis.de).

